# Editorial: State-of-the-art technology and applications in crop phenomics, volume II

**DOI:** 10.3389/fpls.2023.1195377

**Published:** 2023-05-10

**Authors:** Wanneng Yang, John H. Doonan, Xinyu Guo, Xiaohui Yuan, Feng Ling

**Affiliations:** ^1^ National Key Laboratory of Crop Genetic Improvement, National Center of Plant Gene Research, Hubei Hongshan Laboratory, Huazhong Agricultural University, Wuhan, China; ^2^ The National Plant Phenomics Centre, Institute of Biological, Environmental and Rural Sciences, Aberystwyth University, Aberystwyth, United Kingdom; ^3^ Beijing Key Lab of Digital Plant, Research Center of Information Technology, Beijing Academy of Agriculture and Forestry Sciences, Beijing, China; ^4^ College of Plant Protection, Jilin Agricultural University, Changchun, China; ^5^ Key Laboratory for Environment and Disaster Monitoring and Evaluation, Hubei, Innovation Academy for Precision Measurement Science and Technology, Chinese Academy of Sciences, Wuhan, China

**Keywords:** high-throughput phenotyping, AI-driven phenotypic analysis, UAV, hyperspectral, X-ray micro-CT

High-throughput acquisition and analysis of phenotypic data is crucial for plant breeding, as phenotypes are the language of plants and a way for them to express their growth status. We can understand plants and discover their secrets of life through the phenotypes. The crop phenotyping community has put a lot of effort into collecting, processing, and analyzing phenotypic data, which is increasingly considered an important tool for rapidly advancing genetic gain in breeding programs (Zhao et al., 2019). Various types of data have emerged from diverse phenotyping platforms ranging from lab-scale to field-scale, and as a result, various phenotypic data processing approaches have emerged at this historic moment. By integrating knowledge from life science, optics, artificial intelligence, computer science, and engineering, plant phenomics has developed into a cutting-edge discipline, and considerable progress has been made in both phenotyping facilities and methodologies.

To promote the most advanced research progresses in crop phenomics, this Research Topic is prepared and released, which covers new advances in crop phenomics, including phenotyping platforms, methods, and applications. Although various phenotyping platforms have been developed, there is still a great demand for cost-effective phenotyping platform. Deep learning, as a highly effective technology, is applied to various types of images such as RGB, hyperspectral, and CT images to produce highly accurate phenotypic parameters. Meanwhile, the potential of machine learning approaches has also been demonstrated in phenotypic analysis, such as post-harvest quality control, breeding, plant research, and plant response to environmental stress.

## Phenotyping platform design and development

The need for cost-effective phenotyping has been increasingly urgent over the past decade. To make phenotyping platforms more affordable, various aspects such as hardware investment, software solutions, labor cost, and tool sharing have been considered (Reynolds et al., 2019). In particular, the cost of phenotyping hardware is a critical factor in platform design and development. Consequently, some researchers are now directing their attention to developing low-cost phenotyping platforms. A low-cost fluorescence phenotyping platform consisting of four UV lamps, 12 white lamps, and a digital camera was built to evaluate dynamic infection process of Tobacco mosaic virus-green fluorescent protein in tobacco leaves. The performance was evaluated by comparing fluorescence images with RGB images acquired by the system, and the results showed that green fluorescence changes could be identified by the cheap and simple equipment and the non-destructive detection of TMV-GFP was realized (Ye et al.).

In recent years, 3D phenotyping has gained increasing attention in the field due to the ability to generate volumetric information that is not possible with 2D phenotyping. However, the production of 3D structures of plants remains a vital issue that needs to be addressed. Active technologies, such as LiDAR (Light Detection and Ranging), can generate highly accurate 3D geometric information of plants. However, the high cost of hardware investment associated with LiDAR is a significant concern. Alternatively, passive technologies, such as 3D reconstruction methodologies from images, have been investigated due to their low hardware cost. Nevertheless, local personnel costs may be required for data collection, calibration, and processing. At individual plant scale, a miniaturized shoot phenotyping platform, MVS-Pheno V2, has been developed to enable 3D reconstruction of individual plants using multi-view stereo techniques. Based on the MVS-Pheno V1 platform designed for low plant shoots, the MVS-Pheno V2 platform consists of four parts. The hardware is miniaturized to occupy less space, while controlled imaging conditions have been established to avoid the effects of light and wind and ensure high-quality images. Additionally, wireless communication and control have been integrated to avoid cable tangling. The data processing system includes 3D point cloud generation, calibration, and 3D phenotype extraction. To evaluate the performance of the platform, three cultivars of wheat shoots at four different growth stages were used in testing (Wu et al.). Roots are an essential part of all vascular plants that grow underground. Dowd et al. designed and developed “3D root Mesocosms” to enable the visualization and analysis of full-sized plant root architecture in 3-dimensions. The system uses a growth container with an internal volume of 45 ft^3^ (1.27m^3^) that is suitable for large crop and bioenergy grass root systems. The setup only requires an appropriate studio space and a digital camera, and the 3D visualization is created using a photogrammetric reconstruction pipeline. The system was evaluated on root systems of switchgrass, maize, and sorghum to demonstrate its capability for comparing and studying different species.

To study root phenotyping in 2D space, a high-throughput *in situ* root phenotyping platform called the RhizoPot platform was designed and developed with integrated hardware and software. The platform consists of four parts. The RhizoPot cultivated seedlings, while RhizoAuto collected *in situ* root images. A deep learning framework, DeepLabv3++, was used to segment root images, and WinRhizo software was used to obtain dynamic root phenotypes. Root hair phenotypes were analyzed by RhizoComp. This platform allows for the efficient and high-throughput analysis of the dynamic response characteristics of root phenotypes (Zhao et al.). Besides, the platform was used to investigate the effects of high-temperature weather on cotton seedlings’ growth dynamics of the above-ground parts and root phenotype by using images taken by this platform (Fan et al). The RhizoPot platform is a cost-effective solution that can save significant labor costs and offer a general solution for root phenotyping. It can be used for root phenotypic studies and various phenotypic tasks across multiple crops.

In addition to the external phenotype parameters of crops, the internal structure of crops is also a key focus of breeders. Imaging technologies such as CT imaging have entered the field of phenotype research. Despite the requirement for a high initial hardware investment, the use of high-quality equipment can result in promising outcomes. For example, a Micro-CT imaging system was developed to non-destructively acquire CT projection images of passion fruit, followed by 3D model reconstruction (Lu et al.). Comparable accuracy to manual operations was achieved regarding the external traits of passion fruit, while more reliable traits were achieved regarding the internal traits.

## Combination of deep learning and RGB images

Regarding the methods used in plant phenomics, machine learning is one of the most widely used algorithms for data analysis. Among these methods, deep learning is essentially a neural network with three or more layers, allowing it to learn from large amounts of data. The advantages of deep learning, such as its efficient data processing capabilities, automatic feature extraction functions, and high prediction accuracy, make it an important tool for crop phenomics data analysis and prediction.

Crop phenomics generates massive amounts of image data of various types, including RGB images, hyperspectral images, CT images, and microscope images. Object detection, which recognizes and detects different objects in an image to classify them, is a key component of numerous agricultural tasks and applications, such as object counting and phenotyping (Wosner et al., 2021). Filed wheat spikes and rice panicles are the most important agronomic traits associated with yield. Thus, quick, and accurate detection and counting of spikes and panicles has always been one of the most important scientific Research Topics, especially the state-of-the-art performance in object detection and counting brought by deep learning has greatly facilitated progress in the spike and panicle detection and counting. A RetinaNet (SpikeRetinaNet) was trained for spike detection (Wen et al.), and several improvements were made to resolve the issues such as multi-scale feature fusing, efficiency, and occlusion problem.

The YOLOX (You Only Look Once) series of deep learning models have been shown to be powerful and versatile object detection models, offering high accuracy, speed, and flexibility, which make them an attractive option for use in plant phenomics. One of the YOLOX models, YOLO5, has been demonstrated to perform well in detecting and counting small panicles in field rice images under varying illumination and across different rice accessions, even with large image sizes (Wang et al.). In addition to its use in rice panicle detection and counting, the YOLOX deep learning model has been applied to the detection and identification of mature soybean stem nodes, and has been shown to be an effective tool for extracting stem-related phenotypes of mature soybeans. In a comparative analysis with other algorithms, YOLOX achieved a maximum average accuracy (mAP) of 94.36% in detecting soybean stem nodes. To facilitate the identification of soybean structural features, a direct search algorithm was designed (Guo et al.).

In crop phenomics, image segmentation, partitioning each pixel in a given image to provide an accurate representation of the object shapes, have replaced the traditional manual observation and measurement of phenotypic data (Luo et al., 2023). The developed deep learning models, including VGG, FCN, U-Net, SegNet, DeepLab, etc. are often used for conducting pixel-wise segmentation in crop phenomics. In current Research Topic, roots were automatically segmented from the *in situ* root images collected by RhizoPot platform by using DeepLab V3+ network, and it was integrated into the RhizoPot system (Zhao et al.).

Beyond the RGB images, a semantic segmentation model, the U-Net convolution neural network, was implemented on the CT images to distinguish different tissues in the samples (Lu et al.). Supervised image segmentation using a deep learning model usually needs to provide high-quality training sets, which are very labor-intensive. To address this issue, a label generation method based on digital image processing was designed, and the segmentation results with higher accuracy were picked as labels to be used for training.

Vision based classification is also applied in crop phenomics. Aiming at the online real-time identification and classification of tobacco shred types and their actual production in the field, the efficient and accurate identification of different tobacco shred types were urged. An MS-X-ResNet network was constructed by selecting ResNet50 network as the prominent network, and an accuracy of 96.65% was achieved (Niu et al.).

## Combination of hyperspectral imaging and machine learning

Hyperspectral imaging is a rapidly developing method of crop phenomics because it captures both spectral and spatial information. Abiotic, biotic, and quality traits in crops in indoor and outdoor growing conditions can be detected, and phenotyping traits can be generated from a cellular to landscape scale (Sarić et al., 2022). Meanwhile, machine learning algorithms have become an essential tool for hyperspectral image analysis due to their outstanding prediction power, and consequently, in-depth study was developed in the physiological and biochemical research of crops by combining hyperspectral imaging and machine learning.

In terms of maize leaves, estimating the amino acid content can be useful for improving yield. Researchers used the PLSR (Partial Least Square Regression) algorithm to create various models for amino acid content by analyzing the reflectance of all bands, sensitive band ranges, and sensitive bands. The study demonstrated the potential for machine learning and hyperspectral imaging to be combined for genetic sensitivity analysis and variety improvement of maize (Shu et al.) In addition to leaves, seeds are also a focus of phenotypic research using hyperspectral imaging. In the study of wheat seeds, purity was identified as a key factor in the population of hybrid wheat. The transmittance and reflectance spectra provided a better solution for classifying hybrids and female parents than reflectance spectra alone. Specifically, a classification model, Detrend-CARS-PLS-DA based on the PLSR algorithm, was established using the transmittance spectrum combined with a characteristic wavelength-screening algorithm (Zhang et al.).

Deep learning models have demonstrated their power in crop phenotyping. Yu et al. provide an exciting strategy for predicting phenotyping traits of lettuces from spectral reflectance by developing two end-to-end models based on 2D CNN and FCNN, highlighting the potential contribution of combining deep learning models with spectroscopy for phenotype trait quantification. The use of hyperspectral imaging for stress symptom detection has been demonstrated (Lowe et al., 2017). The potential of combining hyperspectral reflectance and deep learning in assessing cotton drought resistance among different genotypes has also been shown. A 1D-CNN was designed and established to screen the spectral information of cotton leaves because it had natural structural consistency with one-dimensional spectral information. A vector with 1024 spectral features was used as the input, and the output layer was the chlorophyll fluorescence parameter Fv/Fm prediction value, which is significant in abiotic plant stress (Guo et al.).

## Machine learning assisted trait analysis

The combination of machine learning and hyperspectral imaging has led to significant achievements, and applying machine learning algorithms to other types of imaging data holds considerable promise. LF-NMR/MRI, a non-destructive and accurate technology for assessing water status, has been widely applied in the fields of food and agriculture. Gu et al. used Principal Component Analysis and LF-NMR imaging to investigate the distribution and dynamics of water in the Xudou 20 soybean cultivar post-germination after culturing plants with varying concentrations of 6-benzylaminopurine (6-BA). In another study, Gao et al. utilized a 50-layer CNN model Residual-Network (ResNet-50) to predict the storage time of Newhall navel oranges based on high-resolution digital microscope images. The study by Feng et al. evaluated the potential of machine learning models to identify biomarkers for subspecies discrimination and yield heterosis prediction in rapeseed, in order to better understand ecotype divergence. Five machine learning algorithms, including discriminant analysis (DCA), random forest (RF), support vector machine (SVM), multilayer perceptron (MLP), and CNN, were utilized for this purpose.

The papers in this Research Topic highlight new advances in phenotyping facilities, methodologies, and analysis that enable the collection of diverse image data ranging from the micro level to the whole-plant level. Phenotyping methodologies and phenotypic analyses, which range from the cellular level to the field scale, utilize various machine learning/deep learning and computer vision algorithms. These papers provide valuable insights into how advanced technologies can be used to measure and analyze crop traits and how this information can be used to improve crop breeding, management, and response to environmental stress. They demonstrate the potential of cutting-edge technologies to revolutionize crop research and production.

## Author contributions

WY initially drafted the manuscript with inputs from other topic editors. All the topic editors read and approved the final manuscript.
